# NF-κB in Gastric Cancer Development and Therapy

**DOI:** 10.3390/biomedicines9080870

**Published:** 2021-07-23

**Authors:** Supattra Chaithongyot, Phatcharida Jantaree, Olga Sokolova, Michael Naumann

**Affiliations:** Institute of Experimental Internal Medicine, Medical Faculty, Otto von Guericke University, 39120 Magdeburg, Germany; supattra.chaithongyot@med.ovgu.de (S.C.); phatcharida.jantaree@med.ovgu.de (P.J.); olga.sokolova@med.ovgu.de (O.S.)

**Keywords:** chemoresistance, gastric inflammation, NF-κB signaling, tumor microenvironment

## Abstract

Gastric cancer is considered one of the most common causes of cancer-related death worldwide and, thus, a major health problem. A variety of environmental factors including physical and chemical noxae, as well as pathogen infections could contribute to the development of gastric cancer. The transcription factor nuclear factor kappa B (NF-κB) and its dysregulation has a major impact on gastric carcinogenesis due to the regulation of cytokines/chemokines, growth factors, anti-apoptotic factors, cell cycle regulators, and metalloproteinases. Changes in NF-κB signaling are directed by genetic alterations in the transcription factors themselves, but also in NF-κB signaling molecules. NF-κB actively participates in the crosstalk of the cells in the tumor micromilieu with divergent effects on the heterogeneous tumor cell and immune cell populations. Thus, the benefits/consequences of therapeutic targeting of NF-κB have to be carefully evaluated. In this review, we address recent knowledge about the mechanisms and consequences of NF-κB dysregulation in gastric cancer development and therapy.

## 1. Introduction

Gastric cancer (GC) is highly prevalent among the gastrointestinal cancers and accounts for the third most leading cause of cancer-related mortality worldwide after lung and liver cancers [1]. The incidence of GC shows geographical variations, with higher rates in East Asian regions and lower rates in North America and North Africa [2,3]. Although the incidence rate of GC is considered to have decreased in past decades, its frequently advanced-stage diagnosis restricts therapeutic options and therefore still leads to high mortality.

GC is a multifactorial disease with heterogeneity in phenotypes, prognoses, and responses to standard chemotherapeutic drugs. GC is characterized by anatomy into two main entities: gastroesophageal junction adenocarcinomas (cardia GCs) and gastric adenocarcinomas (non-cardia GCs). The majority of GC are non-cardia GCs which can be histologically classified based on Lauren classification into intestinal (gland-like structures) and diffuse types (lacks any glandular structures) [4]. Moreover, it can also be classified clinically as early or advanced GC. The risk factors for GC include environmental factors, ethnicity, dietary habits, alcohol consumption, smoking, and, importantly, host genetic factors [5,6]. Although the pathogenesis and development of GC was correlated to multiple factors, a major risk factor is the infection with *Helicobacter pylori*, which was classified as class one carcinogen [7,8].

*H. pylori* is a Gram-negative human pathogen that colonizes the gastric epithelium. Almost half of the world’s population is infected with *H. pylori*, mainly in developing countries [9]. *H. pylori* infection is primarily acquired during childhood and significantly influenced by geographical context, specific living conditions, and familial socioeconomic status. Transmission of *H. pylori* is considered to occur through oral-oral or fecal-oral routes [10].

Despite advances in surgical techniques and the development of a combination of chemotherapy, radiotherapy, and molecular-targeted treatment, the survival rate of patients with GC remains unsatisfactory [11,12,13,14,15]. GC without metastasis can be potentially cured with surgery; however, most patients have an advanced inoperable stage or have recurrent disease after resection. Further research is therefore required to elucidate the molecular mechanisms underlying the tumorigenesis of GC in order to identify novel therapeutic as well as prognostic targets. Here, the NF-κB has a potential role in GC development.

The NF-κB family of transcription factors is ubiquitously expressed and plays an essential role in the regulation of a wide variety of biological processes including cell differentiation, proliferation, survival, and, most importantly, immune responses and inflammation [16]. Five members of the NF-κB family have been identified: RelA, RelB, c-Rel, NF-κB1 (p50), and NF-κB2 (p52), which are bound to each other to form homodimers and heterodimers [17]. In contrast to the other family members, NF-κB1 and NF-κB2 are synthesized as precursors (p105 and p100) which can be processed to p50 and p52, respectively. These five NF-κB family members share a highly conserved 300-amino acid Rel Homology Domain (RHD), which is essential for the dimerization as well as the binding to DNA and interaction with inhibitors of NF-κB (IκBs). In the absence of stimuli, NF-κB dimers predominantly retain in the cytosol by their interaction with IκBs. The IκBs (IκBα, IκBβ, IκBγ, IκBζ, IκBɛ, IκBNS, and Bcl-3) are characterized by ankyrin repeats, which interact with the RDH domains of NF-κB proteins [17].

It has been reported that NF-κB is often upregulated or dysregulated in GC, where it contributes to proliferation, tumor growth, metastasis, and chemoresistance [18,19,20]. As such, components involved in the NF-κB regulation have turned out to be interesting therapeutic targets for the treatment of GC. Here, we review NF-κB signaling in gastric carcinogenesis and putative therapeutic strategies.

## 2. Dysregulation of NF-κB in Gastric Cancer

The dysregulation of NF-κB activation represents an underlying cause of GC development [21]. Ooi et al. [22] developed a genomic taxonomy of GC by using patterns of oncogenic pathways and identified NF-κB signaling as one of the dominant pathways deregulated in GC. Results from other studies indicate that the activation of NF-κB affects gastric carcinogenesis by promoting the activation of genes involved in cell proliferation, suppression of the apoptosis, metastasis, genomic instability, and drug resistance [23,24].

### 2.1. NF-κB Signaling

NF-κB dimers are activated by two main signaling pathways, the classical and the non-canonical pathways [25]. The classical NF-κB pathway becomes activated by diverse stimuli such as interleukin 1β (IL-1β), tumor necrosis factor (TNF), as well as ligands of bacterial origin [25]. Upon stimulation and upstream signaling, IκBα is phosphorylated by a multi-subunit IκB kinase (IKK) complex, consisting of two catalytic subunits (IKKα and IKKβ) and NF-κB essential modulator (NEMO). The phosphorylation of IκBα at two N-terminal serines triggers ubiquitin-dependent IκBα degradation in the 26S proteasome. Subsequently, NF-κB translocates into the nucleus, where it binds to the κB enhancer sequences to induce the activation of specific genes [17].

Interestingly, it has been reported that NF-κB was activated in pathogen infection by ADP-glycero-β-D-manno-heptose (ADP-hep), a key metabolic intermediate in lipopolysaccharide (LPS) biosynthesis [26]. The protein alpha-kinase 1 (ALPK1) and tumor necrosis factor receptor-associated factor (TRAF)-interacting protein with forkhead-associated domain (TIFA) are vital components in response to ADP-hep leading to the activation of classical NF-κB in pathogen infection including *H. pylori*-infected gastric epithelial cells [26,27]. Further, *H. pylori* classical NF-κB activation involves TRAF6, transforming growth factor β kinase 1 (TAK1), and the IKK complex [28].

By contrast, only a small number of stimuli induce the non-canonical NF-κB pathway including ligands of a subset of TNF-receptor superfamily members such as lymphotoxin β (LTβ) receptor, B cell activation factor (BAF), CD40, and receptor activator of NF-κB (RANK) [29,30]. Upon activation of this pathway, NF-κB inducing kinase (NIK) phosphorylates IKKα which phosphorylates carboxy-terminal serine residues of p100, triggering the degradation of the C-term of p100. Further, non-canonical NF-κB signaling in *H. pylori* infection also involves NIK accumulation [31]. The N-terminal part of p100 represents NF-κB2 p52, which translocates with the bound RelB into the nucleus [29].

### 2.2. NF-κB Gene Polymorphisms

One major cause, which affects the NF-κB activity, is represented by polymorphisms in NF-κB genes (Table 1). Here, *NFKB1* (encodes p105 and p50 by alternative splicing) polymorphisms appeared to be associated with GC progression. Single nucleotide polymorphisms of the rs28362491 (located in the promoter region of *NFKB1*), rs230521 (*NFKB1* intron 4), and rs4648068 (*NFKB1* intron 12) have been observed in GC patients [32]. It has been reported that *NFKB1* polymorphism −94 ins/del ATTG (rs28362491) is closely associated with the development of the diffuse type of GC. Furthermore, gastric mucosal inflammation was more severe in *H. pylori*-infected del/del ATTG homozygotes, suggesting that *NFKB1* −94 del/del homozygote may accelerate severe gastric inflammation [33,34]. Along with this observation, Lo et al. [35] provided evidence that polymorphisms of *NFKB1* are associated with susceptibility of GC in aged patients. rs4648068 (A  >  G) polymorphism in the intron region of *NFKB1* was correlated with an increased risk of GC, especially for the lymph node status in Han Chinese population. People with the homozygous GG alleles in rs4648068 strengthened the transcriptional activity of *NFKB1* [36,37]. In addition, NF-κB1 deficiency in mice resulted in invasive GC that reflected the histopathological progression of human intestinal-type gastric adenocarcinoma [38].

p100 encoded by *NFKB2* plays an essential role in many chronic inflammatory diseases. Mice with a homozygous deletion of NF-κB2 had gastric hyperplasia and early postnatal death [40]. In addition, the relative expression level of *NFKB2* mRNA is lower in patients with GC when compared to the control tissue [50]. Mice lacking *NFKB1* (*Nfkb1*−/−) develop gastric atrophy of greater severity than wild-type mice. In contrast, mice lacking the p100/p52 subunit (*Nfkb2*−/−) were protected from developing gastric mucosal lesions [39]. miR-9 has been reported to target NF-κB1 and regulates GC cell growth, suggesting the role of NF-κB1 in human GC pathogenesis [51]. Accordingly, the detection of variations in NF-κB genes could be promising for the prognosis and treatment of GC.

### 2.3. Gene Polymorphisms in NF-κB Signaling Molecules

In addition to genetic alterations of NF-κB genes themselves, aberrantly activated NF-κB signaling molecules have also been associated with gastric carcinogenesis (Table 1). Susceptibility of rs2233408 T/C genotype in the promoter region of *NFKBIA* (gene encoding IκBα) was studied by Wang et al. [41]. They found that this genotype was associated with an increased risk for GC. On the other hand, *NFKBIA* rs2233408 T heterozygote markedly reduced GC risk compared with rs2233408 C homozygote in intestinal-type non-cardiac GC. In line with these findings, *NFKBIA* rs17103265 deletion homozygote was identified as a risk factor for gastric carcinogenesis, especially in southern Chinese populations [42]. Li et al. [43] studied the correlation between three sites of polymorphisms (*NFKB1*, *NFKBIA* rs696 in the 3′-UTR region, and rs2233406 in the promoter region) and the GC risk in the Chinese population. They found that the *NFKBIA* rs696 site was linked with the susceptibility of cardia cancer while *NFKBIA* rs2233406 mutation was associated with the susceptibility of non-cardia cancer, with heterozygous mutations increasing the risk of non-cardia cancer. *IΚBKB* encodes IKKβ, one of the core catalytic subunits of the IKK complex. Single nucleotide polymorphisms in *IΚBKB* have been related to GC. In addition to the evidence that patients with rs2272736 A allele in *IΚBKB* had significantly prolonged overall survival time compared to those with the G allele, AA genotype was shown to have reduced risk of death for GC compared with that associated with the GG/GA genotypes, which was more common in patients with cardiac GC [44].

TNF-induced protein 3-interacting protein 1 (*TNIP1*) encodes an A20-binding protein which plays an important role in the inhibition of NF-κB activation. It has been reported that single nucleotide polymorphisms in the *TNIP1* gene (rs7708392 and rs10036748) were significantly associated with GC risk in the Chinese Han population from Northwest China [45].

Adaptor molecule Myeloid differentiation primary response 88 (MyD88)-induced NF-κB signaling has been related to gastric mucosal damage and carcinogenesis [46] by *MYD88* gene deletions and mutations. Further, MyD88 has been reported to be overexpressed in GC compared with the adjacent non-tumor tissues and its overexpression was correlated with tumor, node, metastasis (TNM) stage and lymph node metastasis. Moreover, silencing of high-mobility-group-protein B1 (HMGB1)/Toll-like receptor (TLR)4/MyD88 signaling by HMGB1 siRNA markedly suppressed gastric cell proliferation, migration, and induced apoptosis through the NF-κB pathway [52]. Interestingly, *MYD88* L265P mutants are constitutively active and capable of signaling to activate NF-κB, signal transducers and activators of transcription 3 (STAT3), and activator protein 1 (AP1) transcription factors, which was observed in gastric mucosa-associated lymphoid tissue (MALT) lymphomas [47].

Receptor interacting serine/threonine kinase 2 (RIPK2), an intracellular kinase that contains a caspase recruitment domain at its carboxy terminus, is a potent activator of NF-κB. RIPK2 was upregulated both at mRNA and protein levels in GC tissues and modulated GC cell proliferation, migration, and apoptosis through the NF-κB signaling pathway [53]. In addition, *RIPK2* single nucleotide polymorphism rs16900627 A > G minor allele was associated with an increased risk for the development of GC, particularly the intestinal type [48].

In the *H. pylori*-infected gastric mucosa, the expression of TLRs was upregulated [54,55,56]. TLR9-1237T/C polymorphism is significantly associated with the development of *H. pylori*-induced premalignant gastric changes by increasing TLR9 transcriptional activity through the activation of NF-κB [49]. In line with these findings, both deficient and excessive expression of TLR4 promotes ethanol-induced gastric mucosal injury by activating the MyD88/NF-κB signaling pathway [57], demonstrating the association of TLR-mediated NF-κB activation and GC development.

To better understand the effect of the mutations reported in NF-κB signaling, further studies are necessary to provide a causal link between NF-κB deregulation and the development of the disease.

### 2.4. Modulation of NF-κB Regulation in Gastric Cancer

Sasaki et al. [58] demonstrated that an increased NF-κB activation as measured by nuclear translocation of RelA correlated with GC invasion and tumor size. Further, knockdown of NF-κB1 and RelA inhibited gastric cell invasion and migration as well as suppressing patient-derived tumors in xenografts [59], suggesting a role of NF-κB in gastric carcinogenesis. NF-κB1 (p105/p50) deficiency, even loss of a single allele, resulted in dysregulated expression of effectors of inflammation, antigen presentation, and immune checkpoints leading to a spontaneous invasive GC in mice [38].

Oncoprotein metadherin (MTDH) was reported to be involved in the activation of the NF-κB signaling pathway [60]. During the tumorigenesis and progression of GC, miR-3664-5P suppressed the proliferation and metastasis of GC by attenuating the NF-κB signaling pathway through targeting MTDH, which was validated in vitro and in vivo [61]. Caspase-associated recruitment domains (CARDs) are involved in apoptosis and inflammation through NF-κB signaling. Kim et al. [62] demonstrated increased CARD6 expression in gastric carcinoma.

Phosphatase of regenerating liver-3 (PRL-3) plays a crucial role in proliferation, metastasis, and angiogenesis. By interaction with repressor/activator protein 1 (RAP1), PRL-3 activates NF-κB signaling through modulating phosphorylation of RelA. Zhang and co-workers [63] proved that PRL-3 promotes GC migration and invasion by positively regulating the NF-κB–hypoxia inducible factor 1 alpha (HIF-1α)–miR-210 axis.

Several other cellular factors, which contribute to the progression of gastric carcinoma through modulating the NF-κB signaling have been described and include Cullin 4A [64], TNF [65], stomach-specific protein gastrokine 1 (GKN1) [66], interleukin 17A [67], IL-1β polymorphisms [68], cytoskeleton protein radixin [69], fibroblast growth factor-inducible 14 (Fn14) [70], inhibitor of growth 4 (ING4) [71], trefoil factor 1 (TFF1) [72], connective tissue growth factor (CTGF) [73], carcinoembryonic antigen-related cell adhesion molecule 19 (CEACAM19) [74], DNA repair protein (Ku) [75], stress protein metallothionein 2A (MT2A) [76], deacetylase sirtuin 1 (SIRT1) [77], oncogenes latent membrane protein 1 (LMP1) and LMP2A [78], microRNAs [79,80,81,82], or spermine oxidase [83].

Overall, several lines of evidence have identified NF-κB as one of the major mechanisms of gastric carcinoma, highlighting the potential of NF-κB of being a therapeutic target as well as a useful prognostic factor in human GC.

## 3. NF-κB-Regulated Genes and Their Relevance for Gastric Cancer Development

The microenvironment of transformed tissue consists of different cell populations, including tumor cells, fibroblasts, endothelial cells, cancer-associated stromal cells, neutrophils, macrophages, which secrete immune response mediators, and effectors in proliferation, cell cycle, apoptosis, and invasion [84,85,86] Certainly, NF-κB transcription factors are widely involved in these processes (Table 2).

### 3.1. Immune Response Mediators

Pro-inflammatory cytokines (IL-6, IL-8, and TNF), cell adhesion molecules CD44 and ICAM-1, and MMPs, e.g., MMP-9, are induced in the epithelium in a NF-κB-dependent manner [117,118]. Immunogenomic analysis has revealed that neutrophils, macrophages, dendritic cells, and eosinophils are abundant in gastritis and further accumulate during progression to atrophic gastritis and GC, where, among others, natural killer T cells, immature B cells, and T follicular helper cells are additionally recruited [119].

In a mice model, an intensive infiltration of the mucosa with neutrophils and macrophages occurs transiently within two days after *H. pylori* infection and remains increased by 2–3 weeks post infection. At that time, the number of T helper cells (CD4 + CD3 + lymphocytes), cytotoxic (CD8+) lymphocytes, and dendritic cells infiltrating the gastric mucosa markedly increases [120]. The leucocytes and lymphocytes support and potentiate the local inflammation and tissue remodeling by producing and responding to the inflammatory mediators, including NF-κB-dependent IL-1β, IL-8, IL-17, C-C motif chemokine ligand 5 (CCL5), CCL28, CCL20, IFNs, C-X-C chemokine ligand 1 (CXCL1—growth-regulated oncogene (GRO-α)), CXCL2-GRO-β/γ, CXCL11, and CXCL10 (IP-10) [119,121]. The production of cytokines is enhanced in people carrying polymorphisms at positions −511 (C > T, rs16944) and −31 (T > C, rs1143627) in the IL-1β gene, and polymorphisms at position −174 (G > C, rs1800795) in the IL-6 gene, which predisposes to GC development [122].

NF-κB drives the expression of anti-inflammatory mediators as well. IL-10 is known to down-regulate the release of pro-inflammatory IL-1β, IL-6, IL-8, TNF, and granulocyte-macrophage colony-stimulating factor (GM-CSF) in monocytes and lymphocytes. Decreased production of IL-10 due to an ATA haplotype of −1082/+819/+592 polymorphism in the IL-10 gene can lead to a stronger inflammation in *H. pylori*-infected patients and might be associated with a risk of ulcer disease or non-cardia GC development [123,124].

The immune response primed by antigen-presenting cells and potentiated by lymphocytes, especially by pro-inflammatory Th1 and Th17, is actually required for the tissue protection against microbial agents by controlling their proliferation and dissemination. T regulatory (Treg) cells and Th2 lymphocytes perform an anti-inflammatory function [125]. In manifest GC, where an infectious agent does not play a significant role anymore, interactions between several cell subsets and cytokines amplify or suppress growth of the tumor and shape immune responses against tumor cells. Despite a recent progress in research, the link between inflammatory cytokines and the transition through gastritis–chronic atrophic gastritis–metaplasia–dysplasia–GC remains not entirely clear.

Increased IL-8 mRNA levels in the gastric mucosa correlate with diffuse-type GC, despite showing no relation with survival rate [87]. Kido et al. [88] found that the IL-8 level in human gastric carcinomas correlated significantly with the depth of invasion, venous invasion and lymphatic invasion, and low survival rate [88]. Proinflammatory cytokine IL-17 has been shown to be positively associated with GC [121]. Interestingly, both IL-8 and IL-17 stimulate an expression of the NF-κB target gene *MMP-9* in human gastric cancer cell lines MKN-1 and AGS, respectively, which enhances cell migration and invasion [67,89].

The overproduction of IL-1β in a transgenic mouse model causes a stepwise progression of gastric dysplasia to GC. This cytokine-stimulated recruitment of macrophages, granulocytes, and dendritic cells to the mucosa supports an inflammatory environment by the expression of NF-κB target genes IL-6, TNF, and chemokine CXCL12 (aka SDF1), and promotes an oncogenic transformation by suppressing T- and B-cells [90].

Macrophages in tumors can exhibit M1 (immunostimulating) or M2 (immunosuppressing) functional characteristics. The M2 macrophages (tumor-associated macrophages, TAMs) promote GC. An increase above the median M1/M2 ratio in gastric tumors has been suggested as a positive independent predictor of survival [126]. The number of TAMs correlates with gastric tumor stage, serosa invasion, and lymph node metastasis [91]. The mechanism of macrophage polarization towards M2 phenotype is not entirely clear. However, it involves interaction with gastric cancer-derived mesenchymal stromal cells (MSCs) and is mediated by IL-6 and IL-8, as shown in a mice xenograft model and cell co-culture experiments [92]. TAMs can promote the invasion and migration of GC cells in co-culture experiments by stimulating the expression of NF-κB-regulated genes (COX2, MMP9, and VEGF) [91,92,93]. TAMs themselves express VEGF and VEGF-C in a NF-κB dependent manner, thus impacting angiogenesis in the tissue [91].

It has been shown that TAMs-released TNF and IL-6 influence the expression of programmed-death ligand 1 (PD-L1) in gastric tumor cells via NF-κB and STAT3 signaling, thereby promoting the proliferation of GC cells [127]. PD-L1 on tumor cells is the ligand for T-cells-expressed programmed death 1 (PD-1), and it is one of the so-called immune checkpoint molecules, which can dump cytotoxic T-cell response towards tumors [95]. The human PD-L1 promoter encompasses potential NF-κB binding sites, but in addition to gene transcription, NF-κB signaling pathways can participate in the regulation of PD-L1 level via regulating other mediators [128]. In GC, the data regarding the role of PD-L1 are contraposing. The expression of PD-L1 in resected tissue (43.6% of samples) was related to a less advanced stage, intestinal type, and well/moderately differentiated adenocarcinoma, as well as to a better disease-free survival of patients with GC [94]. In contrast, Junttila et al. [95] demonstrated that the recruitment of CD3+ and CD8+ immune cells in tumors was associated with an improved overall survival, but PD-L1 expression in these tumors was associated with poor prognosis for patients with GC. Whether the immune checkpoint molecules are promising targets for the immunotherapy of gastrointestinal cancers is still under investigation [129].

### 3.2. iNOS and COX2

In epithelial cells and macrophages, the NF-κB transcription factors contribute to the up-regulation of inducible nitric oxide synthase (iNOS, NOS2) and cyclooxygenase-2 (COX-2). iNOS and COX-2 catalyze the production of nitric oxide (NO) and prostaglandin E2 (PGE2), respectively, in humans, in mice models, or in gerbil models of infection and gastric cancer [93,96,130]. NO and PGE2, similar to cytokines, act as paracrine inflammatory mediators. They potentiate the infiltration of macrophages in stomach tissue and promote an inflammatory environment [96,97,98]. Further, PGE2 and NO promote tissue healing via eliminating infectious agents, increasing tissue microcirculation and cell restitution [99]. However, their long-lasting effect contributes to oxidative stress, DNA damage, increased expression of DNA methyltransferases, e.g., DNA methyltransferase 3 (DNMT3). This leads to promoter methylation of tumor suppressors, e.g., O6-methylguanin-DNA-methyltransferase (MGMT), cannabinoid receptor 1 (CNR1), protection of telomeres 1 (POT1), ataxia-telangiectasia mutated (ATM), and cadherin-1 (CDH1), and accelerates the turnover of epithelial cells, increasing the mutagenesis rate in inflamed tissue [100,101,102,103].

It has been believed for more than 20 years that iNOS and COX-2 contribute to a gradual progress of gastric carcinogenesis [104,105,106,107]. Thus, their chemical targeting remains an object of investigation [131,132]. It has been shown that two weeks of treatment with the selective COX-2 inhibitor Rofecoxib resulted in the increase of caspase-3 cleavage, decline of B-cell lymphoma 2 (Bcl-2), and survivin expression in tumors, and in parallel, decreased gastrin level in the plasma of GC patients [133]. Further, in patients with intestinal metaplasia, two years of treatment with Rofecoxib down-regulated levels of methylation in tissue. An inhibition of COX-2 in combination with DNMT using celecoxib and decitabine synergistically inhibited gastric tumor growth in vitro and in vivo [102]. In COX-2-overexpessing GC patients, the combination of celecoxib and conventional chemotherapy increased the progression-free overall survival [134].

### 3.3. Effectors in Proliferation, Cell Cycle, Apoptosis, and Invasion

Via transcription of the E3 ubiquitin ligase MDM2, NF-κB influences the p53 stability, and thereby cell proliferation, and via induction of *Hif1α*, it contributes to the cell response to hypoxia, which is relevant to tumorigenesis [135]. In GC cells, the inhibition of NF-κB reduces the expression and activation of STAT3 [136]. Both transcription factors are known to contribute to GC development and progression [108]. NF-κB1 (p105/p50) deficiency, even loss of a single allele, resulted in aberrant JAK-STAT1 signaling and dysregulated expression of effectors of inflammation, antigen presentation, and immune checkpoints leading to a spontaneous invasive gastric cancer in mice [38].

A significantly higher expression level of NF-κB/RelA and its target genes, *c-myc* and *cyclinD1*, was also detected in intestinal-type gastric carcinoma [109]. In addition, small RNAs such as miR-223-3p, miR-18a-3p, and miR-4286 have been reported in gastric cancer cells and tissues and linked to *H. pylori*-induced NF-κB signaling and cellular proliferation as well as gastric carcinogenesis [80,111].

Infection with *H. pylori* affects paracrine loops between parietal cells, gastrin-secreting G cells, histamine-secreting ECL cells, and somatostatin-secreting D cells [137,138]. In *H. pylori*-infected human gastric tissue, the expression of ATP4A, a subunit of H, K-ATPase, is suppressed in a T4SS-dependent and NF-κB-dependent manner [137]. By intervening in proton pump expression and decreasing acidity, *H. pylori* might facilitate its own survival in the stomach. This will increase bacterial load and promote immune responses and tissue damage. The suppression of H, K-ATPase intensifies gastrin production, which stimulates the oxyntic mucosa, and contributes to its atrophy, promoting further hypergastrinemia and ECL cell hyperplasia [104,138]. There is an indication that gastrin and released factors by ECL cells promote the proliferation of gastric cells, including cell precursors, which is also relevant for gastric cancer pathogenesis [138]. Further, cytokines contribute to these functional disorders [139].

In tumor cells exposed to infection and/or cytokines, NF-κB has been shown to regulate transcription of genes with pro-survival functions. Immunohistochemistry (IHC) analysis demonstrated a significant positive correlation between NF-κB and antiapoptotic phosphoprotein dopamine and cAMP-regulated phosphoprotein 32,000 Da (DARPP-32) expression levels in GC tissues as well as in *H. pylori*- and TNF-treated cells [140]. IL-1β-induced NF-κB regulates expression of retinoid x receptor α, a member of the nuclear receptor superfamily involved in proliferation, differentiation, apoptosis, and metabolism [141]. IL-1β-induced NF-κB activate expression of HNF4α, a member of the nuclear receptor superfamily [110]. HNF4α is involved in many human malignancies via regulating Wnt/β-catenin, NF-κB, STAT3, and TGFβ signaling pathways to increase cell migration and invasion and decrease apoptosis [142]. In gastric cells, HNF4α stimulates the expression of IL-1 receptor 1, which further amplifies IL-1β/NF-κB signaling and directs sustained inflammation and GC. In clinical samples, HNF4α and IL-1 receptor 1 levels are increased in *H. pylori*-induced gastritis and reach their highest levels in GC [110]. NF-κB induces miR-425, which negatively regulates phosphatase and tensin homolog (PTEN) expression, thereby promoting the proliferation of GC cells [112]. NF-κB directly regulates the expression of NADPH oxidase organizer 1 (Noxo1), a component of NADPH oxidase 1 (NOX1), in TNF-stimulated GC cells. This effect is associated with increased ROS levels in mouse models for gastritis and GC. NOX1/ROS signaling is suggested to promote proliferation of sex determining region Y (SRY)-box 2 (SOX2)-positive gastric stem cells, which leads to gastritis-associated metaplastic hyperplasia [113].

NF-κB contributes to the regulation of the invasive properties of human gastric carcinoma cells by stimulating the production of, e.g., MMP-9 in response to IL-1β or MMP-2 and MMP-12 in response to TNF-CXCL1/CXCL2-S100A8/9 activation [143,144,145]. In atrophic gastritis and GC tissue, RelA has been suggested to potentiate the expression of the long-noncoding RNA HOX transcript antisense RNA (HOTAIR), which promotes cell motility and invasion through targeting Wingless int 1 (Wnt)/β-catenin and poly-r(C)-binding protein (PCBP) [146]. In addition, NF-κB is involved in the up-regulation of Snail1, which leads to E-cadherin reduction in GC tissue [114]. A ubiquitously expressed CXC chemokine CXCL12, which usually stimulates the migration of monocytes and T-lymphocytes, induces NF-κB pathway-dependent expression of its receptor CXC chemokine receptor 4 (CXCR4) and epidermal growth factor receptor (EGFR) in GC cell lines, which further activate IKKα/ß and RelA and increase cell migration ability [147].

Human telomerase reverse transcriptase (hTERT) which upregulates Cdx2 through NF-κB signaling showed the promotion of intestinal metaplasia. Previous studies found an increased expression of NF-κB and hTERT in dysplasia, intestinal metaplasia, and GC [115,116]. The activation of NF-κB may lead to hTERT expression, and thus, enhance telomerase activity, which represents an important step in carcinogenesis.

## 4. Therapeutic Targeting of NF-κB in Gastric Cancer

**Figure 1:**
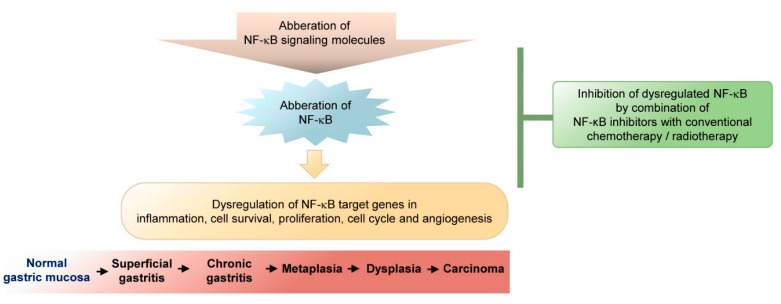
NF-κB dysregulation in the pathogenesis of gastric cancer. Prerequisites for the progression to gastric carcinoma development could include superficial and chronic gastritis, metaplasia, and dysplasia. The impact of NF-κB on gastric carcinoma development could be affected by the aberration of NF-κB signaling molecules or the NF-κB transcription factors themselves and the dysregulation of NF-κB target genes. The various NF-κB target genes are involved in a variety of cellular processes including inflammation, cell survival, proliferation, cell cycle, or angiogenesis. Therapeutic approaches for gastric carcinoma need the development of inhibitors which interfere with the dysregulated NF-κB system in combination with conventional chemo- or radiotherapy.

NF-κB dysregulation contributes to the development of chronic inflammation and cancer progression in heterogeneous tumor cell populations (epithelial and immune cells). A high total and nuclear abundance of RelA in gastric tumors has been shown to correlate with advanced stage and poor patient survival [114,148]. However, in human stage IV gastric carcinoma, RelA expression was found to decrease, which was predictive of a better efficacy of treatment with paclitaxel/LV5Fu2 or FOLFOX [149]. In contrast, Lee et al. [150] reported that high NF-κB activity in early-stage gastric carcinoma correlated with better prognosis. Further, it has been shown that NF-κB can be induced following treatment of GC cells with cytotoxic agents, e.g., docetaxel, cisplatin, or 5-fluoruracil (5-FU) [151,152,153]. Here, NF-κB collaborates with other signaling pathways triggered by genotoxic stress [154], and up-regulates transcription of pro-survival genes, including cyclin D1, Bcl-2, and survivin, which contribute to chemoresistance [155]. Thus, consequences/benefits of NF-κB therapeutic targeting have to be carefully evaluated (Figure 1).

Investigations of the tumor microenvironment have shown that different tumor cell populations respond differently to anti-cancer therapy. For example, 3,3’-diindolylmethane (DIM), a bioactive compound derived from indole-3-carbinol of *Brassica* food plants, inhibited growth of cancer cells but induced expression of tumor-related factors CCL-2, IL-6, and IL-8 in mesenchymal stem cells (MSCs). The conditioned medium of DIM-treated MSCs promoted the proliferation, invasion, and migration of GC cells in vitro and tumor growth in vivo. This effect was mediated by DIM-induced expression of an E3 ubiquitin ligase component beta-transducin repeat containing E3 ubiquitin protein ligase (β-TrCP) in MSCs, which caused degradation of IκBα and activation of NF-κB. Thus, MSCs support anti-cancer effects of the drug on cancer cells [156]. These data suggest the usage of a combination of NF-κB inhibitors with conventional chemotherapeutics or radiotherapy. Indeed, in a mice GC model, intraperitoneal administration of RelA siRNA or nafamostat mesilate (FUT-175), an inhibitor of serine proteases and NF-κB, potentiated Paclitaxel effects leading to a reduction of peritoneal metastasis and increasing survival [157,158].

The cholesterol-lowering drug simvastatin enhanced the apoptotic effects of capecitabine through suppression of NF-κB-regulated genes in mice xenografts [155]. The combined therapy of paclitaxel with the sesquiterpene lactone parthenolide, which inhibited phosphorylation of NF-κB, efficiently suppressed the peritoneal dissemination, and prolonged survival time in a mice model of GC [159].

A range of inhibitors has been developed for targeting NF-κB signaling including TNF receptors, IKKs, IκBα, or RelA/p50. Several molecules have reached stage 3 clinical trials and have been approved for the treatment of many tumors, but very few for gastric cancer therapy [160,161]. For example, among several promising 26S proteasome inhibitors, bortezomib has been approved for anti-tumor therapy of multiple myeloma, diffuse large B-cell lymphoma, colorectal cancer, and thyroid carcinoma. NF-κB inhibition by bortezomib is achieved via suppression of proteasomal degradation of IκBα and suppresses survival of GC cancer cell lines in cell culture and in subcutaneous transplants in mice [162]. Despite showing promising results in experimental models, bortezomib has not been used for therapy of human GC. Interestingly, disulfiram, a well-known drug for treatment of chronic alcoholism, can inhibit the 26S proteasome and NF-κB activity and demonstrate anti-tumor activities [163].

High expression of IKKβ and NEMO has been reported to correlate with shorter overall survival in GC [164]. Accordingly, IKKβ-knockout mice exhibit increased apoptotic cell death in gastric mucosal epithelium and decreased IL-1a secretion, and tumor formation in response to N-methyl-N-nitrosourea [165]. Inhibitors of NF-κB signaling, including IKKα/β inhibitors BAY11-7082 and BAY11-7085 demonstrated anti-proliferative (e.g., via suppressing cyclin A and cyclin-dependent kinase 2 (CDK-2) expression), pro-apoptotic (e.g., via down-regulation of Bcl-2, up-regulation of Bcl-2-associated X protein (Bax)), and anti-invasive abilities in GC cell lines and in xenograft models [166,167].

A number of natural compounds demonstrated NF-κB inhibiting and anti-tumor activity in cell and animal models [168]. For example, the carotenoid lycopene, in addition to its antioxidant properties, inhibits transcriptional activity of NF-κB, down-regulates expression of IL-1β, IL-6, TNF, and COX2, and activates caspase-3 and caspase-9 in AGS cells [169]. Phyto-compound curcumin and its chemical analogs exert anti-GC effects by downregulating NF-κB activity [170,171]. Polyphenols capsaicin, quercetin, resveratrol, epigallocatechin-3-gallate, etc. also exhibit anti-tumor and anti-inflammatory properties via affecting COX and NF-κB [161]. Several hormones and vitamins, e.g., melatonin and vitamin E, reduce the production of ROS in GC cells in part via diminishing NF-κB activity and MMPs expression in tumor-associated cell populations [172,173].

Some reports have suggested that nonsteroidal anti-inflammatory drugs (NSAIDs) and COX2 inhibitors reduce risk of GC [174,175]. Sulindac, NO-aspirin and NO-naproxen as well as COX-2 inhibitor SC236 suppressed the IKKs and NF-κB [172,176]. On the other hand, some NSAIDs (e.g., indomethacin) can cause mucosa damage and even activate NF-κB [177,178]. Thus, the pro and contra of their usage for cancer prevention or therapy are still a matter of debate [176].

Members of the NF-κB signaling pathways have not been explored so far for molecular targeted therapy in GC. At present, molecular therapy of GC is restricted to targeting the human epidermal growth factor receptor 2 (HER2) by trastuzumab [179,180].

In the last decade, it became clear that NF-κB as a driver of a number of cellular processes plays an important role in GC development. Despite intensive investigations towards targeting immune cells, their molecular mediators, and effectors, there is still no established immunotherapeutic strategy for treatment of GC. However, the inhibitors of PD-L1 reached stage 3 clinical trials and demonstrated promising anti-tumor activity in patients with PD-L1-positive GC [181,182,183].

## 5. Conclusions

NF-κB controls a variety of cellular processes comprising inflammation, proliferation, and anti-apoptosis. Therefore, it is not surprising that the studies reviewed here demonstrate an important role of NF-κB in the carcinogenesis of gastric tumors. There are manifold alterations in NF-κB expression and NF-κB signaling molecules by gene polymorphisms resulting in dysregulated NF-κB target genes. Further, regulation of NF-κB within the different cell populations is diverse which contributes to a complex molecular interplay in the tumor micromilieu. Thus, analyzing the pleiotropic functions of NF-κB in detail would be of significant interest to anticipate the outcome for the control of cellular functions in different cell populations. Although the NF-κB system provides promising biomarkers for diagnostics and therapeutic targeting in cancer patients, therapeutic targeting of NF-κB could also elicit opposing effects. Deciphering the diverse outcomes of NF-κB activity depending on the cellular context and relative to specific stimuli will enable the design of therapeutic targeting strategies to treat gastric cancer without overall cytotoxicity.

## Figures and Tables

**Table 1:** Polymorphisms in NF-κB genes and genes of NF-κB signaling molecules.

Gene Name	Genetic Aberration	Comments	References
*NFKB1*	SNP_rs230521	observed in GC patients	[32]
	SNP_rs28362491 (−94 ins/del ATTG)	associated with diffuse GC, accelerate severe gastric inflammation	[33,34]
	SNP_rs4648068	increased risk of GC	[36,37]
	homozygous deletion	invasive GC, gastric atrophy in mice	[38,39]
*NFKB2*	homozygous deletion	gastric hyperplasia, early postnatal death	[40]
		suppressed in gastric mucosal lesions	[39]
*NFKBIA*	SNP_rs2233408 T/C homozygote	GC susceptibility	[41]
	SNP_rs2233408 T heterozygote	reduced GC risk in intestinal-type non-cardiac GC	
	SNP_rs17103265	risk factor for gastric carcinogenesis	[42]
	SNP_rs696	cardia GC susceptibility	[43]
	SNP_rs2233406	non-cardia GC susceptibility	
*I* *ΚBKB*	SNP_rs2272736 A homozygote	prolonged overall survival time	[44]
*TNIP1*	SNP_rs7708392	associated with GC risk	[45]
	SNP_rs10036748		
*MYD88*	deletion, mutation	gastric mucosal damage, carcinogenesis	[46]
	L265P mutant	observed in gastric mucosa-associated lymphoid tissue (MALT) lymphomas	[47]
*RIPK2*	SNP_rs16900627	increased risk of intestinal GC	[48]
*TLR9*	SNP_rs5743836 (−1237 T/C)	associated with *H. pylori*-induced GC	[49]

**Table 2:** NF-κB-regulated genes and their relevance for gastric cancer development.

NF-κB Regulated Genes	Comments	References
*IL-8*	correlates with diffuse-type GC	[87]
	correlates with depth of invasion, venous and lymphatic invasion, low survival rate, enhances cell migration and invasion	[88]
*IL-17*	positively associates with GC, enhances cell migration and invasion	[67,89]
*IL-1β*	promotes gastric dysplasia to GC	[90]
*COX2*, *MMP9*, *VEGF*	TAMs induce *COX2*, *MMP9*, *VEGF* expression, promote invasion/migration in GC	[91,92,93]
*PD-L1*	relates to a less advanced stage, intestinal type GC	[94]
	associates with poor prognosis for GC patients	[95]
*NO*, *PGE2*	potentiates the infiltration of macrophages in stomach tissue, promotes an inflammatory environment	[96,97,98]
	promotes tissue healing via eliminating infectious agents, increasing tissue microcirculation and cell restitution	[99]
	accelerates turnover of epithelial cells, increasing the mutagenesis rate in inflamed tissue	[100,101,102,103]
*iNOS*, *COX-2*	contributes to a gradual progress of gastric carcinogenesis	[104,105,106,107]
*STAT3*	contributes to GC development and progression	[108]
*c-myc*, *cyclinD1*	high expression in intestinal-type GC	[109]
*HNF4α*	HNF4α overexpression correlates with sustained inflammation and GC	[110]
*miR-223-3p*, *miR-18a-3p*, *miR-4286*	expression in gastric cancer cells and tissues, links to proliferation and gastric carcinogenesis	[80,111]
*miR-425*	promotes proliferation of GC	[112]
*Noxo1*	associates with gastritis and GC	[113]
*Snail1*	downregulation of E-cadherin in GC tissue	[114]
*hTERT*	promotes intestinal metaplasia	[115,116]

## Data Availability

Not applicable.

## Abbreviations

Activator protein 1	AP1
3,3’-diindolylmethane	DIM
ADP-glycero-β-D-manno-heptose	ADP-hep
Ataxia-telangiectasia mutated	ATM
B cell activation factor	BAF
B-cell lymphoma 2	Bcl-2
Bcl-2-associated X protein	Bax
Beta-transducin repeat containing E3 ubiquitin protein ligase	β-TrCP
Cadherin-1	CDH1
Cag pathogenicity island	CagPAI
Cannabinoid receptor 1	CNR1
Carcinoembryonic antigen-related cell adhesion molecule 19	CEACAM19
Caspase-associated recruitment domains	CARDs
Chemokine (C-X-C motif) ligand 1	CXCL1
Chemokine (C-X-C motif) ligand 2	CXCL2
Connective tissue growth factor	CTGF
CXC chemokine receptor 4	CXCR4
CXC chemokine receptor 4	CXCR4
CXC motif chemokine ligand 11	CXCL11
Cyclin-dependent kinase 2	CDK-2
Cyclooxygenase-2	COX-2
Deacetylase sirtuin 1	SIRT1
DNA methyltransferase 3	DNMT3
DNA repair protein	Ku
Dopamine and cAMP-regulated phosphoprotein 32,000 Da	DARPP-32
Epidermal growth factor receptor	EGRF
Epstein–Barr virus (EBV) latent membrane protein 2	LMP2A
Fibroblast growth factor-inducible 14	FN14
Gastric adenocarcinomas	non-cardia GCs
Gastric cancer	GC
Gastric mucosa-associated lymphoid tissue	MALT
Gastroesophageal junction adenocarcinomas	cardia GC
Gastrokine 1	GKN1
Granulocyte-macrophage colony-stimulating factor	GM-CSF
Growth-regulated oncogene	GRO-α
Growth-regulated protein beta	GRO-β
Growth-regulated protein gamma	GRO-γ
HOX transcript antisense RNA	HOTAIR
Human epidermal growth factor receptor 2	HER2
Human telomerase reverse transcriptase	hTERT
Hypoxia inducible factor 1 alpha	HIF-1α
Immunohistochemistry	IHC
Immunohistochemistry analysis	ICH
Inducible nitric oxide synthase	iNOS, NOS2
Inhibitor of growth 4	ING4
Interleukin 1b	IL-1b
IκB kinase	IKK
Lipopolysaccharide	LPS
Lymphotoxin β	LTβ
Lymphotoxin β	LTβ
Matrix metallopeptidase 9	MMP9
Mesenchymal stromal cells	MSCs
Mucosa-associated lymphoid tissue	MALT
Myeloid differentiation primary response 88	MyD88
NADPH oxidase 1	NOX1
NADPH oxidase organizer 1	Noxo1
NF-κB essential modulator	NEMO
NF-κB inducing kinase	NIK
NF-κB1	p50
NF-κB2	p52
Nitric oxide	NO
Non-cardia gastric adenocarcinoma	NCGC
Nonsteroidal anti-inflammatory drugs	NSAIDs
O6-methylguanin-DNA-methyltransferase	MGMT
Oncogenes latent membrane protein 1	LMP1
Oncoprotein metadherin	MTDH
Open reading frames	ORFs
p21-activated kinases	PAKs
Phosphatase and tensin homolog	PTEN
Phosphatase of regenerating liver-3	PRL-3
Poly r(C)-binding protein	PCBP
Programmed death 1	PD-1
Programmed-death ligand 1	PD-L1
Prostaglandin E2	PGE2
Protection of telomeres 1	POT1
Protein alpha-kinase 1	ALPK1
Reactive oxygen species	ROS
Receptor activator of NF-κB	RANK
Receptor interacting serine/threonine kinase 2	RIPK2
Rel homology domain	RHD
Repressor/activator protein 1	RAP1
Sex determining region Y (SRY)-box 2	SOX2
Signal transducers and activators of transcription 3	STAT3
Stress protein metallothionein 2A	MT2A
T regulatory	Treg
TNF-induced protein 3-interacting protein 1	TNIP1
Toll-like receptors	TLRs
TRAF-interacting protein with forkhead-associated domain	TIFA
Transcription factor nuclear factor kappa B	NF-κB
Transforming growth factor b kinase 1	TAK1
Trefoil factor 1	TFF1
Tumor necrosis factor receptor-associated factor	TRAF
Tumor necrosis factor	TNF
Tumor-associated macrophages	TAMs
Type IV secretion system	T4SS
Vascular endothelial growth factor	VEGF
